# On BER Performance of EBPSK-MODEM in AWGN Channel

**DOI:** 10.3390/s100403824

**Published:** 2010-04-14

**Authors:** Lenan Wu, Man Feng

**Affiliations:** Institute of Information Science and Engineering, Southeast University, Nanjing 210096, China; E-Mails: wuln@seu.edu.cn

**Keywords:** Ultra Narrow Band (UNB), Extended Binary Phase Shift Keying (EBPSK), Rice distribution, Bit Error Rate (BER)

## Abstract

In order to satisfy the higher and higher demand for communication systems, an Extended Binary Phase Shift Keying (EBPSK) system with very high spectra efficiency has been proposed. During the research, a special kind of filters was found, which can amplify the signal characteristics and remove utmost noise, *i.e.*, at the point of the phase jumping corresponding to code “1”, produce the amplitude impulse much higher than code “0”, therefore, the aim of our study was to analyze the BER performance of the impacting filter assisted EBPSK-MODEM. Considering the receiver filtered “0” and “1”signal with Rice amplitude distribution, just having different mean values, so the BER performance of EBPSK is deduced based on the classic detection theory, and compared with the traditional BPSK modulation both in spectra efficiency and in BER performance, which lays the theoretical foundation for the feasibility of Ultra Narrow Band communications based on EBPSK modulation.

## 1. Introduction

With the development of the information society, there is higher and higher demand for communication systems. Therefore, how to transmit the information faster and better has become very important. Recently, high efficiency modulation technologies are receiving the attention by many researchers, especially Ultra Narrow Band (UNB) [1,2] communication with its outstanding transmission ability in the wireless communication field. The concept of UNB communication, first proposed by H. R. Walker, can obtain very high data rates and high spectra efficiency, even higher than traditional channel capacity. Consequently, many researchers begin to pay more attention to this “break” performance, and the extension to Shannon’s channel capacity equation has been proposed [3].

In wireless sensor networks [4], sensor nodes are typically powered by batteries with a limited lifetime, and even though energy-scavenging mechanisms can be adopted to recharge batteries through solar panels, piezo-electric or acoustic transducers, energy is still a limited resource and must be used judiciously, so efficient use of the sensor node battery’s energy is an important aspect of sensor networks. Therefore, many researchers have paid attention to this problem and proposed many energy management schemes [5–7].

During the research, we found that as a kind of UNB system, the Extended Binary Phase Shift Keying (EBPSK) [8] modulation, proposed by Wu *et al*., has better bandwidth efficiency [9], high data rates, attractive energy efficiency [10], and can greatly save energy for sensor nodes. However, the research on its BER performance just remains in the simulation stage and has no detailed theoretical derivations. So this paper will discuss this problem, which will provide the theoretical support for simulation results, and lay the foundation for the feasibility and rationality of UNB system.

The rest of this paper is organized as follows. In Section 2, a scheme for EBPSK- MODEM is introduced, of which the detailed derivation of BER performance is given, and also the SNR improvement performance is analyzed in Section 3. Then, Section 4 collects some simulation results, and conclusions are given in Section 5.

## 2. EBPSK Modulation and Demodulation

### 2.1. The Definition of EBPSK Modulation

EBPSK modulation [8] is defined as follows:

(1) $$\begin{array}{l} g_{0}(t)=A\;\text{sin}\;w_{c}t,0\leq t<T\\ g_{1}(t)=\{\begin{array}{cc} A\;\text{sin}(w_{c}t+\theta ), & 0\leq t<\tau ,0\leq \theta \leq \pi ,\\ A\;\text{sin}\;w_{c}t, & 0\leq \tau \leq t<T, \end{array} \end{array}$$

where *g*_0_(*t*) and *g*_1_(*t*) is modulation waveform corresponding to bit “0” and bit “1”, respectively, *T* is the bit duration, *τ* is the phase modulation duration, and *θ* is the modulating angle. Obviously, if *τ* = *T* and *θ* = *π*, Eq.(1) degenerates to:

(2) $$g_{0}(t)=A\;\text{sin}\;w_{c}t,\;\;\;\;g_{1}(t)=-A\;\text{sin}\;w_{c}t,\;\;\;\;0\leq t<T$$

**Figure 1.**
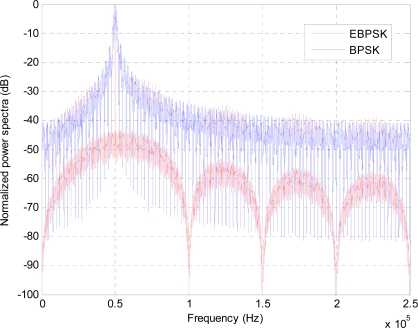
PSD of the BPSK and EBPSK modulations, all carrier peaks are normalized at 0dB.

It is just the classical Binary Phase Shift Keying (BPSK) modulation, so is named as the Extended BPSK. Figure 1 depicts the normalized the power spectral density (PSD) of the typical BPSK of (2) and the EBPSK of (1) at the same bit rate *R*, where bit duration *T* = 20*T_c_* = 20*2*π* / *w_c_*, the phase modulation duration *τ* for EBPSK modulation lasts one period of carrier, *i.e.*, *τ* = *T_c_* = 2*π* / *w_c_*, the modulation angle *θ* = *π*, the amplitude *A* = 1, and the carrier frequency *f_c_* = *w_c_* / 2*π* = 50*KHz*,

### 2.2. The Demodulation of EBPSK Signals

As we know, if $A=\sqrt{\frac{2E_{b}}{T}}$, the bit error ratio (BER) of the optimal BPSK receiver is calculated by:

(3) $$P_{e-\mathrm{BPSK}}=Q\left(\sqrt{\frac{2E_{b}}{N_{0}}}\right)=\frac{1}{\sqrt{2\pi }}\int_{\sqrt{\frac{2E_{b}}{N_{0}}}}^{\infty }\text{exp}\left(-\frac{u^{2}}{2}\right)\mathrm{du}$$

where *E_b_* is the signal energy used for transmitting one bit, and *N*_0_ is the ***PSD*** of Addition Gaussian White Noise (AWGN).

**Figure 2.**
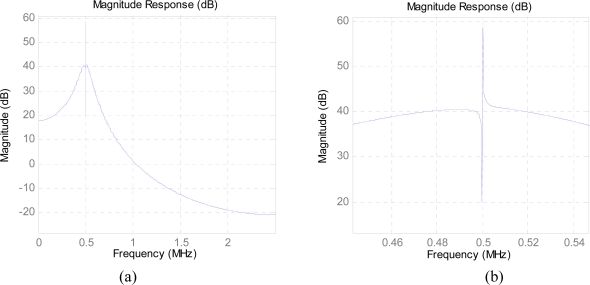
(a) The amplitude-frequency response of an “impacting filter”. (b) The zoomed figure of (a).

**Figure 3.**
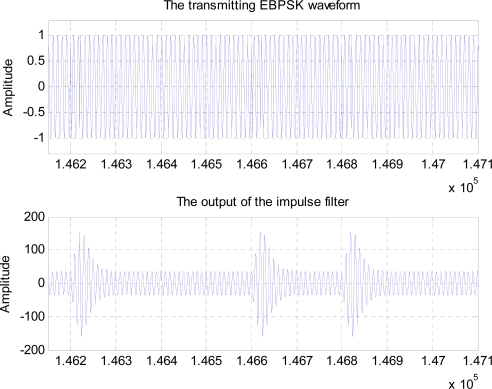
The output of the impacting filter to the EBPSK modulated input.

However, if the same optimal BPSK receiver using traditional matched filter was utilized as the EBPSK demodulator, the BER performance would be much poorer since the difference in EBPSK modulated waveforms corresponding to “0” and “1” is very tiny and hard to detect, although in Figure 1 more centralized PSD of the EBPSK appears. Therefore a special infinite impulse response (IIR) filter as given in Figure 2 is used in EBPSK receiver to produce high impulse at the phase jumping points *τ* of bit 1s, so as to transform phase modulation into amplitude changes [10]. And by this way it amplifies the signal characters as much as possible and removes utmost noise. Figure 3 depicts the response of this filter to EBPSK modulated signals. Obviously, a simple amplitude detector followed would perform the demodulation of EBPSK signals because of the existence of high impulse in coded 1 s. Therefore, we gave this kind of filter the name of “impacting filter” [10].

## 3. BER Performance of EBPSK Receivers

### 3.1. The Derivation of a BER Expression

In this section, the BER performance of the impacting filter based EBPSK receiver is analyzed in an AWGN channel.

The EBPSK modulated waveform corresponding to “0” is a pure sine wave, after passing the AWGN channel and the special impacting filter at the receiver, then the envelope *r*_0_ of the filter output is with Rice distribution [11], whose probability density function (pdf) [12] is as follows:

(4) $$p(r_{0})=\frac{r_{0}}{\sigma^{2}}\text{exp}\left(-\frac{r_{0}^{2}+A_{0}^{2}}{2\sigma^{2}}\right)\cdot I_{0}\left(\frac{r_{0}A_{0}}{\sigma^{2}}\right)$$

where *A*_0_ is the amplitude of the filter output, *σ*^2^ the noise variance, and *I*_0_(*z*) the zero-order modified Bessel function, defined as follows:

(5) $$I_{0}(z)=\sum_{n=0}^{\infty }\frac{z^{2n}}{2^{2n}\cdot n!\cdot n!}$$

A similar analysis can also be done aiming at code “1”. If we only consider those periods with phase jumping during *τ*, the jumping information can be transformed into parasitic amplitude information after receiving filter, *i.e.*, producing the higher amplitude impulse. At this time, its envelope *r*_1_ still is Rice distribution, the corresponding pdf is:

(6) $$p(r_{1})=\frac{r_{1}}{\sigma^{2}}\text{exp}\left(-\frac{r_{1}^{2}+A_{1}^{2}}{2\sigma^{2}}\right)\cdot I_{0}\left(\frac{r_{1}A_{1}}{\sigma^{2}}\right)$$

This derivation of the BER is based on the special and linear impacting filter as given in Figure 2, which can produce the amplitude impulse at the point of the phase jumping corresponding to code “1” that is much higher than the background of code “0”, *i.e.*, *A*_1_ > *A*_0_. Let *A*_1_ = *A*_0_ + Δ*A* = *A*_0_ + *kA*_0_ and *k* > 0

Therefore, assuming that code “0” and “1” be transmitted with equal probability and the decision threshold be *U_T_*, then the BER of the EBPSK system should be:

(7) $$\begin{array}{ll} P_{\mathrm{e-EBPSK}} & =\frac{1}{2}[P(1|0)+P(0|1)]\\ & =\frac{1}{2}\left[\int_{U_{T}}^{\infty }\;p(r_{0}){\mathrm{dr}}_{0}+\int_{-\infty }^{U_{T}}\;p(r_{1}){\mathrm{dr}}_{1}\right]\\ & =\frac{1}{2}\left[\int_{U_{T}}^{\infty }\frac{r_{0}}{\sigma^{2}}\text{exp}\left(-\frac{r_{0}^{2}+A_{0}^{2}}{2\sigma^{2}}\right)\cdot I_{0}\left(\frac{r_{0}A_{0}}{\sigma^{2}}\right){\mathrm{dr}}_{0}+\int_{-\infty }^{U_{T}}\frac{r_{1}}{\sigma^{2}}\text{exp}\left(-\frac{r_{1}^{2}+A_{1}^{2}}{2\sigma^{2}}\right)\cdot I_{0}\left(\frac{r_{1}A_{1}}{\sigma^{2}}\right){\mathrm{dr}}_{1}\right]\\ & =\frac{1}{2}[1-F_{0}(u_{T})+F_{1}(u_{T})]\\ & =\frac{1}{2}\left[1+Q_{1}\left(\frac{A_{0}}{\sigma },\frac{U_{T}}{\sigma }\right)-Q_{1}\left(\frac{(1+k)A_{0}}{\sigma },\frac{U_{T}}{\sigma }\right)\right] \end{array}$$

where *F*_0_(*x*) and *F*_1_(*x*) is cumulative distribution function of Ricean random variable *r*_0_ and *r*_1_, respectively, and *Q*_1_(*a,b*) is Marcum’s fuction, defined as follows:

(8) $$Q_{1}(a,b)=e^{-(a^{2}+b^{2})/2}\sum_{k=0}^{\infty }{\left(\frac{a}{b}\right)}^{k}\;I_{k}(\mathrm{ab})$$

### 3.2. Calculation of the Parameters in the BER Formula

We will now discuss in detail how to ascertain the parameters *A*_0_, *k*, *σ*^2^ and *U_T_* in the BER formula (7) in this subsection.

(1) The amplitude *A*_0_ is evaluated as follows:

In EBPSK modulation and via Fourier transform:

(9) $$g_{0}(t)=A\;\text{sin}\;w_{c}t\overset{F}{↔}G_{0}(w)=A\frac{\pi }{j}[\delta (w-w_{c})-\delta (w+w_{c})]$$

Let the frequency response of the filter be *H*(*w*), then the signal filtered in frequency domain can be written as:

(10) $$\begin{array}{ll} Y_{0}(w) & =G_{0}(w)\cdot H(w)=A\frac{\pi }{j}[\delta (w-w_{c})-\delta (w+w_{c})]\cdot H(w)\\ & =A\frac{\pi }{j}\cdot H(w_{c})\cdot \delta (w-w_{c})-A\frac{\pi }{j}\cdot H(-w_{c})\cdot \delta (w+w_{c}) \end{array}$$

So the waveform in time domain is:

(11) $$\begin{array}{ll} y_{0}(t) & =F^{-1}[Y_{0}(w)]=\frac{A}{2j}\left[H(w_{c})\cdot e^{{\mathrm{jw}}_{c}t}-H(-w_{c})\cdot e^{-{\mathrm{jw}}_{c}t}\right]\\ & =\frac{A}{2j}\left[|H(w_{c})|\cdot e^{j(w_{c}t+\varphi_{1})}-|H(-w_{c})|\cdot e^{-j(w_{c}t-\varphi_{2})}\right] \end{array}$$

where *φ*_1_ = ∠*H*(*w_c_*), *φ*_2_ = ∠*H*(−*w_c_*). The impacting filter has conjugate zero points and poles, so *φ*_1_ = −*φ*_2_, and |*H*(*w_c_*)| = |*H*(−*w_c_*)|, then the received signal after filter is as follows:

(12) $$y_{0}(t)=|H(w_{c})|\cdot A\;\text{sin}\;(w_{c}t+\varphi_{1})$$

So *A*_0_ = *A* · |*H*(*w_c_*)|, *A*_1_ = (1+*k*) · *A* · |*H*(*w_c_*)|, and in accordance with Eq.(3), let $A=\sqrt{\frac{2E_{b}}{T}}$. Finally we get:

(13) $$A_{0}=\;|H(w_{c})|\cdot \sqrt{\frac{2E_{b}}{T}}$$

(2) The variance *σ*^2^ is evaluated as follows:

Let the ***PSD*** of the input AWGN be *N*_0_ / 2, measured in ***Watt/Hz***, then the variance *σ*^2^ at the output of the filter should be:

(14) $$\sigma^{2}=\frac{N_{0}}{2\pi }\int_{0}^{\infty }{\left|H(w)\right|}^{2}\mathrm{dw}=N_{0}\int_{0}^{\infty }{\left|H(f)\right|}^{2}\mathrm{df}$$

(3) The threshold *U_T_* is evaluated as follows.

**Figure 4.**
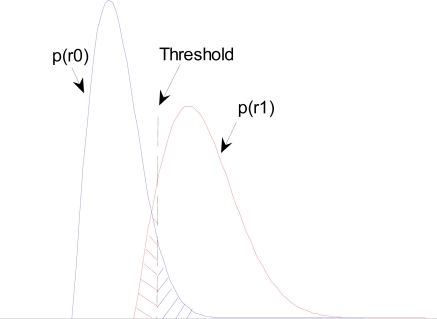
The sketch map of the detection performance by pdf.

Based on the binary detection model, the sketch map of the detection performance illustrated by pdf is shown in Figure 4, where shaded area indicates the BER. *P*(1|0) and *P*(0|1) represent shaded area at the right and left of the threshold, respectively. Obviously, when threshold increases, *P*(1|0) will decrease, while *P*(0|1) will increase, and *vice versa*. Therefore, in order to obtain a minimum BER, the optimal decision threshold *U_T_* should be located at the intersection point of curve *p*(*r*_0_) and *p*(*r*_1_), *i.e.*, the theoretical minimum BER should be equal to half of the area in the intersection part of curve *p*(*r*_0_) and *p*(*r*_1_).

(4) The parameter *k* is evaluated as follows:

According to Figure 4, the larger is the parameter k, the further is the distance of the curve *p*(*r*_0_) and *p*(*r*_1_), *i.e.*, the smaller is the area of the shaded part, *i.e.*, the smaller is the system BER. Therefore, we hope to choose the larger k value in order to improve BER performance.

Among all the parameters discussed above, how to select *k*, the increment in amplitude at the beginning of bit “1” after filtering, needs improving:

Conceptually, the maximum of *kA*_0_ could reach the peak, say ±20*dB*, which is formed by a very narrow frequency selector and trap locating at the center of impacting filter as shown in Figure 2. In practice, the peak of *kA*_0_ might be limited by filter design and waveform selection (such as *τ*, *T* and *θ*), or even the status of channel, which still needs revealing. In fact, as an example given in Figure 3, where the peak of *kA*_0_ is about two times of its background, so at this situation, we may roughly get the estimation as *k* = 2.

### 3.3. Calculation of SNRs

In this subsection we will calculate the various signal to noise ratios (SNRs) so as to evaluate the EBPSK-MODEM.

(1) *SNR* at the input of the impacting filter

Similar as Eq.(14), let the ***PSD*** of the input AWGN be *N*_0_ / 2, then from the autocorrelation function $R\left(\tau \right)=\frac{N_{0}}{2}\cdot \delta \left(\tau \right)$ we get its variance as following:

(15) $${ɛ}^{2}=R(0)\overset{F}{↔}\frac{1}{2\pi }\int_{-\infty }^{\infty }\frac{N_{0}}{2}\cdot \delta (0)\cdot e^{\mathrm{jwt}}\mathrm{dw}=\frac{N_{0}}{2}$$

where the *N*_0_ / 2 is measured in ***Watt*** since it is the average ***power*** of the input noise, although in numerical value it is the same as its ***PSD***.

Let $A=\sqrt{\frac{2E_{b}}{T}}$ again, the average power of the signal at the input of the impacting filter is:

(16) $$\frac{A^{2}}{2}=\frac{E_{b}}{T}=E_{b}\cdot R_{b}$$

which results in the *SNR* calculation at the input of the impacting filter as below:

(17) $${\mathrm{SNR}}_{\mathrm{in}}=\frac{A^{2}}{2\cdot \varepsilon^{2}}=\frac{E_{b}\cdot R_{b}}{N_{0}/2}$$

where *N*_0_ / 2 is the average ***power*** of input noise.

(2) SNR at the output of the impacting filter

The average SNR at the output of the impacting filter for bit “0” during *T* (and in theory for bit “1” during *T* − *τ* also) is:

(18) $${\mathrm{SNR}}_{0}=\frac{1}{2}A_{0}^{2}/\sigma^{2}=\frac{E_{b}}{N_{0}T}\cdot \frac{{\left|H(w_{c})\right|}^{2}}{\int_{0}^{\infty }{\left|H(f)\right|}^{2}\mathrm{df}}=R_{b}\cdot \frac{E_{b}}{N_{0}}\cdot \frac{{\left|H(f_{c})\right|}^{2}}{\int_{0}^{\infty }{\left|H(f)\right|}^{2}\mathrm{df}}=\frac{E_{b}\cdot R_{b}}{N_{0}\cdot B_{\mathrm{neq}}}$$

where *f_c_* is the carrier frequency, *E_b_* is the average energy for transmission of one bit, *R_b_* = 1/*T* is the bit rate because both the BPSK and the EBPSK is binary modulation, so *R_b_* · *E_b_* is the average signal power transmitted, *N*_0_ / 2 is still the ***PSD***, and:

(19) $$B_{\mathrm{neq}}=\int_{0}^{\infty }|H(f){|}^{2}\mathrm{df}/{\left|H(f_{c})\right|}^{2}$$

is the noise equivalent bandwidth of filters [13].

Therefore, the gain in *SNR* of impacting filter for bit “0” should be:

(20) $$G_{0}=\frac{{\mathrm{SNR}}_{0}}{{\mathrm{SNR}}_{\mathrm{in}}}=\frac{E_{b}\cdot R_{b}}{N_{0}\cdot B_{\mathrm{neq}}}/\frac{E_{b}\cdot R_{b}}{N_{0}/2}=\frac{1}{2B_{\mathrm{neq}}}$$

From Eq. (20) it is clear that *B_neq_* is the SNR “controlling factor” for bit “0” determined by the shape of frequency response of the impacting filter used.

Similarly as Eq.(18), the ***peak*** SNR at the output of the impacting filter for bit “1” during its beginning is given as follows:

(21) $${\mathrm{SNR}}_{1}=\frac{A_{1}^{2}}{\sigma^{2}}=\frac{A_{0}^{2}\cdot {\left(1+k\right)}^{2}}{\sigma^{2}}=2{\mathrm{SNR}}_{0}+\frac{A_{0}^{2}\cdot (2k+k^{2})}{\sigma^{2}}>{\mathrm{SNR}}_{0}$$

And the gain in *SNR* of impacting filter for bit “1” should be:

(22) $$G_{1}=\frac{{\mathrm{SNR}}_{1}}{{\mathrm{SNR}}_{\mathrm{in}}}=\frac{1}{B_{\mathrm{neq}}}+\frac{A_{0}^{2}\cdot (2k+k^{2})}{2E_{b}R_{b}\cdot \int_{0}^{\infty }{\left|H(f)\right|}^{2}\mathrm{df}}$$

## 4. Simulation

**Figure 5.**
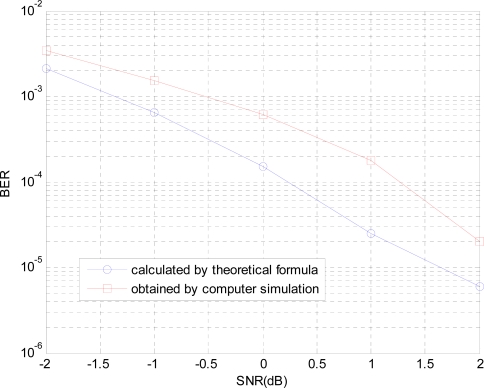
The BERs comparison between calculated by theoretical formula and obtained by computer simulation.

In this section, in order to verify the correctness of theoretical analysis, we compare the BER results calculated by the theoretical formula with one obtained by computer simulation, as in Figure 5, where system parameters in simulation are selected as: *f_c_* = 930*KHz*, *N* = 20, *K* = 2, *A* = *B* = 1, and *θ* = *π*. The paper chooses the receiver filter having three conjugate poles and one zero point, where the detail filter parameters is as ref. [10].

According to the above results, the theoretical result and simulation result just have 1 dB difference in order to obtain the same BER performance, which is caused by the detection method and the choosing of the decision threshold in the simulation.

## 5. Discussion and Conclusions

(1) The *P_e−BPSK_* given in (3) is an ideal and optimum value already, while the *P_e−EBPSK_* given in (7) is far from giving an optimal EBPSK receiver and still depends on the filter design. In other words, it leaves potential for EBPSK receivers to improve. For example, from the Figure 4 we can imagine:

- With the increasing of *k*, the peak distance of *p*(*r*_0_) and *p*(*r*_1_) becomes large, so the shaded area decreases, *i.e.*, the minimum BER decreases.
- With the decrease of *σ*^2^, although the peak distance of *p*(*r*_0_) and *p*(*r*_1_) remains unchanged, their shapes are narrowing, so the shaded area, or the minimum BER, decreases also.

Therefore, under certain conditions, the BER performance of the EBPSK modulation will outperform or be inferior to that of the BPSK modulation, and the spectral efficiency of the EBPSK system is much higher than BPSK.

(2) Substituting *k* = 2 into (21) we obtain an astonishing estimation as *SNR*_1_ = 18*SNR*_0_, which implies that as long as we can obtain larger value of *k* by optimizing the impacting feature of the filter, the BER performance of the EBPSK system can be further improved.

To sum up, aimed at a UNB system, the EBPSK-MODEM, this paper has discussed the importance of the special impacting filter, deduced the BER formula of EBPSK system, analyzed the SNR improvement performance of impacting filter, discussed the choice of optimum decision threshold, and analyzed the reason or possibility for EBPSK to outperform BPSK both in spectral efficiency and in BER performance. Research on these special filters is underway, and a detailed introduction will be given in forthcoming papers.
